# Efficacy evaluation of probiotics combined with prebiotics in patients with clinical hypothyroidism complicated with small intestinal bacterial overgrowth during the second trimester of pregnancy

**DOI:** 10.3389/fcimb.2022.983027

**Published:** 2022-10-06

**Authors:** Yingqi Hao, Yajuan Xu, Yanjie Ban, Jingjing Li, Bo Wu, Qian Ouyang, Zongzong Sun, Miao Zhang, Yanjun Cai, Mengqi Wang, Wentao Wang

**Affiliations:** Department of Obstetrics and Gynecology, The Third Affiliated Hospital of Zhengzhou University, Zhengzhou, China

**Keywords:** breath test, hypothyroidism, intestine, pregnancy, therapy

## Abstract

**Objective:**

To explore the effect of probiotics combined with prebiotics on clinical hypothyroidism during pregnancy combined with small intestinal bacterial overgrowth.

**Methods:**

(1) In total, 441 pregnant women were included in this study. A total of 231 patients with clinical hypothyroidism during the second trimester of pregnancy and 210 normal pregnant women were enrolled in the lactulose methane-hydrogen breath test. The positive rate of intestinal bacterial overgrowth (SIBO), gastrointestinal symptoms, thyroid function and inflammatory factors were compared between the two groups by chi-square test and two independent sample t-test. (2) SIBO-positive patients in the clinical hypothyroidism group during pregnancy (n=112) were treated with probiotics combined with prebiotics based on conventional levothyroxine sodium tablets treatment. The changes in the methane-hydrogen breath test, gastrointestinal symptoms, thyroid function and inflammatory factors were compared before treatment (G0) and 21 days after treatment (G21) by chi-square test and paired sample t test.

**Results:**

(1) The positive rates of SIBO in pregnant women in the clinical hypothyroidism group and control group were 48.5% and 24.8%, respectively. (2) The incidence of abdominal distention and constipation in the clinical hypothyroidism group was significantly higher than that in the control group, and the risk of abdominal distention and constipation in SIBO-positive pregnant women was higher than that in SIBO-negative pregnant women. (3) The serum levels of hypersensitive C-reactive protein (hsCRP), IL-10, IL-6, TNF-α, low-density lipoprotein (LDL), total cholesterol (TC), free fatty acids (FFAs) and apolipoprotein B (ApoB) in the hypothyroidism group during pregnancy were higher than those in the control group. (4) After 21 days of probiotics combined with prebiotics, the incidence of pure methane positivity in the methane-hydrogen breath test in the G21 group was significantly reduced, and the average abundance of hydrogen and methane at each time point in the G21 group was lower than that in the G0 group. (5) The incidence of constipation in the G21 group was significantly lower than before treatment. (6) The levels of serum TSH, hsCRP, IL-6, TNF-α, TC and LDL in pregnant women after probiotics combined with prebiotics were lower than those before treatment.

**Conclusion:**

Probiotics combined with prebiotics are effective in the treatment of pregnant patients with clinical hypothyroidism complicated with SIBO, providing a new idea to treat pregnant patients with clinical hypothyroidism complicated with SIBO.

## Introduction

Thyroid disease is one of the most common endocrine diseases in women of childbearing age, and the incidence of clinical hypothyroidism during pregnancy is 0.3%-0.5% (Casey and T.D.M.a.J.Q, 2020), which can cause adverse outcomes, such as neurodevelopmental disorders in children and fetal growth restriction, and has a significant impact on the growth and development of offspring (Ge et al., 2020; Kerver et al., 2021). In addition, the clinical manifestations of hypothyroidism are not specific and sometimes cannot be distinguished from common symptoms or signs of pregnancy, such as fatigue, constipation, weight gain, edema and dry skin (Casey and T.D.M.a.J.Q, 2020).

The basic concept of small intestinal bacterial overgrowth (SIBO) is that the colonization level of intestinal microorganisms is reduced and the balance of the bacterial community is significantly changed (Pimentel et al., 2020). One of the risk factors is reduced gastrointestinal motility, and hypothyroidism is related to changes in gastrointestinal motility, so hypothyroidism may lead to SIBO (Bohinc Henderson, 2021). The diagnosis of SIBO is based on the lactulose breath test (LBT), which has the advantages of being non-invasive, convenient, sensitive, accurate, and reproducible (Pimentel et al., 2020). Probiotics are active in the small intestine, and prebiotics provide nutrients for probiotics. A combination of both may produce a certain energy effect. In the small intestine, bacterial overgrowth, inflammatory bowel disease, irritable bowel syndrome and other diseases have considerable effects, such as inhibiting bacterial translocation and intestinal mucous membrane barrier function and reducing inflammation (Hamasalim, 2016; Markowiak and Śliżewska, 2017).

To further explore a new idea for the treatment of patients with clinical hypothyroidism complicated with intestinal bacterial overgrowth during pregnancy, this study used probiotics (bifidobacteria tetrad live tablets) combined with prebiotics (polysaccharide fiber powder) to treat patients with clinical hypothyroidism complicated with positive intestinal bacterial overgrowth during pregnancy and evaluated its efficacy.

## Materials and methods

### Experimental subjects

A total of 442 pregnant women who received perinatal care at the Obstetrics Clinic of the Third Affiliated Hospital of Zhengzhou University from July 2020 to December 2021 were included, including 231 pregnant women with clinical hypothyroidism during pregnancy and 210 pregnant women with normal pregnancy. All subjects signed informed consent forms, and the study was approved by the Ethics Committee.

The inclusion criteria for this study were age > 18 years old and < 35 years old, thyroid function in the second trimester met the reference range of clinical hypothyroidism established by the Laboratory of the Third Affiliated Hospital of Zhengzhou University (TSH > 4.32mIU/L and FT4 < 9.77pmol/L, commercial kit (Roche, Shanghai, China)). And the patients used levothyroxine sodium tablets regularly to control thyroid function.

The exclusion criteria included pregnant women with positive thyroid peroxidase antibody; used probiotics, prebiotics, antibiotics and other drugs affecting intestinal flora in the past three months; multiple pregnancies and artificial impregnation; gestational diabetes mellitus, gestational hypertension, systemic lupus erythematosus, thyroid dysfunction before pregnancy and other complications affecting the endocrine system and immune system.

### Experimental method

A total of 231 pregnant women with clinical thyroidism during pregnancy were selected as the Hypothyroidism Group, and 210 pregnant women with normal thyroidism and no pregnancy complications were selected as the Control Group. Fasting blood was taken to detect thyroid function, inflammatory factors and lipid levels.

SIBO-positive patients in the clinical hypothyroidism group during pregnancy (n=112) were included in the further experimental study to explore the changes in parameters of probiotics (bifidobacterium quadrupectin viable tablets) combined with prebiotics (polysaccharide fiber powder) before treatment (G0) and 21 days after treatment (G21). The methane-hydrogen breath test was performed again after 21 days of treatment. Fasting blood was taken to detect thyroid function, inflammatory factors and lipid levels. The dosage of levothyroxine sodium tablets was appropriately adjusted according to the thyroid function and the patient’s tolerance after 21 days of treatment. The dosage of levothyroxine sodium tablets at G0 and G21 was recorded respectively.

Participants were asked not to change their daily eating habits during the study and to avoid consuming foods or drugs containing probiotics or fermented products.

After 21 days of treatment, patients with persistent positive SIBO were treated with berberine based on continued treatment with probiotics combined with prebiotics until SIBO turned negative, and subsequent treatment was no longer included in the study.

### Lactulose methane-hydrogen breath test

Before examination, patients should make proper preparations, such as avoid hydrogen-producing foods, such as dairy products, soy products, wheat flour products and high-fiber vegetables within 24 hours before exhalation. Rice, meat and eggs are edible. At least 12 hours before exhalation on an empty stomach, they were allowed to drink a small amount of water, the empty stomach should be checked on that day and teeth should be brushed first to avoid bacteria in the mouth affecting the results. Patients should be awake and quiet during exhalation, eliminate all beverages, and avoid chewing gum and a smoking environment.

On the morning of the examination day, subjects blew for the first time on an empty stomach. After blowing for the first time, they immediately drank the medicine (lactulose 15 g+warm water 50 ml) in one swallow. After drinking the medicine, they started timing, blew for the second time 20 min later, blew for the third time 20 min after the second time, etc.

### Diagnosis of SIBO

The diagnostic criteria were based on the literature support of the North American consensus and the definition of Breath Tracker SC(QuinTron, USA), a lactulose methane-hydrogen breath test instrument (1) Intestinal bacterial overgrowth is diagnosed as a baseline increase of ≥20 ppm within 90 minutes from the beginning of substrate administration (2) methane concentration ≥10 ppm at any time point is considered to be positive for SIBO (3) if hydrogen and methane concentrations do not reach the above values within 90 minutes of breath test, the sum of the two values is higher than the sum of fasting hydrogen baseline values, and methane concentration exceeds 15 ppm, SIBO is considered positive (Rezaie et al., 2017). In this study, 20-minute intervals were used to reduce the false positive rate because Asian populations have shorter blinding times than Western populations and lactulose reduces the time of mouth blindness (Gwee et al., 2010; Rezaie et al., 2017).

### Gastrointestinal symptoms

(1) Abdominal distension: the following two items must be included: ① Abdominal distension occurs at least 3 days in the last 3 months; ②The diagnosis of functional dyspesia(FD), Irritable Bowel Syndrome(IBS)or other functional gastro intestinal disorders(FGID)is not achieved. The past 3 months meet the diagnostic criteria, and symptoms must appear at least 6 months before diagnosis (Schmulson and Chang, 2011) (2). Constipation: two or more of the following six items (1): in more than a quarter (25%) of the time in the appearance of exertion; ② more than 25% of the time blocking or difficult defecation; ③anus rectum obstruction > 25% (4); incomplete defecation, time in defecation > 25%; ⑤ need manual help defecation time >25%; ⑥defecation < 3 times per week (Jani and Marsicano, 2018) (3); diarrhea: increased stool frequency (more than 3 times per day) and changes in stool properties (mushy liquid) (Schiller et al., 2017).

### Probiotics

In this experiment, a Bifidobacterium quadruple living tablet (Sienkang, National drug approval: S20060010) was used, which is composed of intestinal probiotics and is a compound preparation. The main ingredients include infant Bifidobacterium, Lactobacillus acidophilus, Enterococcus faecalis and Bacillus cereus. Usage: 1.5 g, 3 times a day.

### Prebiotics

Polysaccharide fiber powder (Risikon®, production license number: SC13061011200721) is a dietary supplement made of various dietary fiber complexes, and the main components include inulin, ice threosaccharide, microcrystalline cellulose, and oat fiber. Usage: 5 g each time, 3 times a day.

### Statistical analysis

The statistical analysis software used in this study was SPSS 26.0 (IBM Corp. Released 2019. IBM SPSS Statistics for Windows, Version 26.0. Armonk, NY: IBM Corp). The measurement data with a normal distribution are described as the mean ± standard deviation, and the measurement data with a nonnormal distribution are described as the median and quartile. Paired sample T test and independent sample T test were used for statistical analysis of quantitative data, and the chi-square test (Pearson chi-square and likelihood ratio) was used for statistical analysis of categorical variables. P<0.05 indicated a statistically significant difference.

## Results

**Figure 1:**
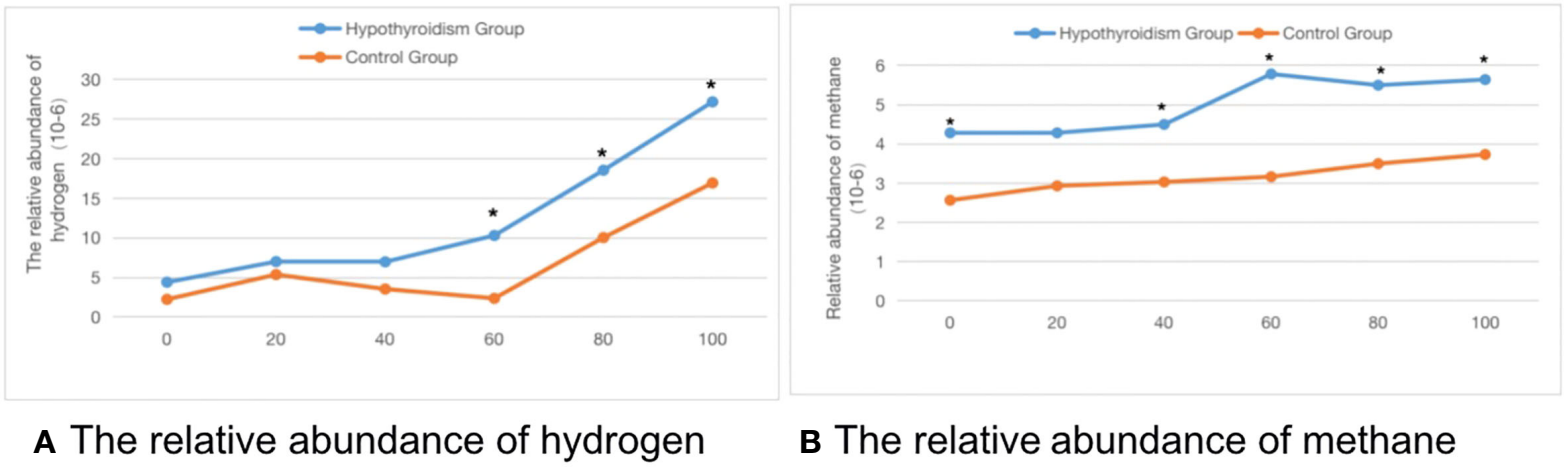
The time-abundance curve of hydrogen and methane in the control group and pregnant clinical hypothyroidism group. **(A)** The relative abundance of hydrogen. **(B)** The relative abundance of methane. *indicates statistical significance.

Comparison of the SIBO positive rate between the two groups: As shown in **Table 1**, the SIBO positive rate in the clinical hypothyroidism group and the control group during pregnancy was 48.5% and 24.8%, respectively. The positive rate of pure methane was 18.6%, which was significantly higher than that of the control group (P < 0.05). The average abundance of hydrogen and methane at each time point in the methane-hydrogen breath test of pregnant women in the clinical hypothyroidism group was higher than that in the control group, there were significant differences in hydrogen at 60, 80 and 100 time points and methane at 0, 40, 60, 80 and 100 time points (**Figure 1**).

**Table 1:** Comparison of the SIBO positive rate, pure hydrogen positive rate, pure methane positive rate and hydromethane positive rate between the clinical hypothyroidism group and the control group during pregnancy.

methane-hydrogen breath test	Control Group N = 210	Hypothyroidism Group N = 231	p
SIBO+ (%)	52 (24.8)	112 (48.5)	<0.001
hydrogen+ (%)	37 (17.6)	57 (24.7)	0.071
methane+ (%)	10 (4.8)	43 (18.6)	<0.001
hydrogen+methane+ (%)	5 (2.4)	12 (4.8)	0.098

p: values of patients in the control group and pregnant clinical hypothyroidism group.

p< 0.05 indicates statistical significance.

Comparison of clinical symptoms between the two groups. As shown in **Table 2**, the incidence of abdominal distention and constipation in pregnant women in the clinical hypothyroidism group was 29.4%, higher than that in the control group. The probability of no obvious symptoms was 25.5%, which was significantly lower than that of the control group (P < 0.05). The incidence of abdominal distention and constipation in SIBO-positive patients was significantly higher than that in SIBO-negative patients during pregnancy.

**Table 2:** Comparison of gastrointestinal symptoms between the hypothyroidism group and the control group during pregnancy.

		diarrhea (n/%)	abdominal distension (n/%)	constipation (n/%)	No obvious symptoms (n/%)
Control Group		27 (12.9)	43 (20.5)	33 (15.7)	107 (51.0)
Hypothyroidism Group		36 (15.6)	68 (29.4)	68 (29.4)	59 (25.5)
P_c-H_		0.306	0.03	0.001	<0.001
Control Group	SIBO+	10 (19.2)	11 (21.2)	9 (17.3)	5 (9.6)
	SIBO-	27 (17.1)	19 (12.0)	23 (14.6)	106 (67.1)
P_c_		0.725	0.103	0.632	<0.001
Hypothyroidism Group	SIBO+	9 (8.0)	24 (21.4)	63 (56.3)	16 (14.3)
	SIBO-	8 (6.7)	5 (4.2)	24 (20.2)	82 (68.9)
P_H_		0.702	<0.001	<0.001	<0.001

pc-H: P values of gastrointestinal symptoms in the control group and pregnant clinical hypothyroidism group.

pc:P values of gastrointestinal symptoms in SIBO-positive and –negative patients in the control group.

pH: P values of gastrointestinal symptoms in patients with SIBO-positive and SIBO-negative clinical hypothyroidism during pregnancy.

p<0.05 indicates statistical significance.

The basic information and clinical indicators of the subjects were compared as follows:In **Table 3**, there were no significant differences in age, height, weight, BMI, IL-2, IL-4, TG, HDL or ApoA1 levels between the two groups (P >0.05). The levels of TSH, hsCRP, IL-10, IL-6, TNF-α, TC, LDL, FFA and ApoB in the pregnancy hypothyroidism group were higher than those in the control group (P <0.05). In **Table 4**, there was no significant difference in the levels of TSH and FT4 between SIBO positive and SIBO negative patients with clinical hypothyroidism during pregnancy (P > 0.05), while the dose of levothyroxine sodium tablets in SIBO positive patients was significantly higher than that in SIBO negative patients (P < 0.05).

**Table 3:** Comparison of general data between pregnant women with clinical hypothyroidism during pregnancy and pregnant women in the control group.

parameters	Control Group (n = 210)	Hypothyroidism Group (n = 231)	F	P value
Age (years)	30.80 ±4.18	30.94 ± 4.76	3.118	0.744
High (cm)	161.60 ± 4.41	161.42 ± 3.96	4.247	0.659
Weigh (kg)	60.75 ± 8.30	60.08 ± 5.22	46.230	0.302
BMI (kg/cm2)	23.33 ± 3.53	23.08 ±2.18	41.548	0.372
FT4 (pmol/L)	13.21 ± 1.56	11.61 ± 1.79	0.032	<0.001
TSH (mIU/L)	1.74 ± 0.90	2.48 ± 1.59	65.704	<0.001
hsCRP (mg/L) TC (mmol/L) TG (mmol/L) HDL (mmol/L) LDL (mmol/L) FFA (mmol/L) ApoA1 (g/L) ApoB (g/L) IL-2 IL-10 IL-6 IL-4 TNF-α	2.71 ± 1.93 5.34 ± 1.11 2.45 ± 1.05 2.14 ± 0.49 3.13 ± 0.74 0.33 ± 0.13 2.34 ± 0.36 1.11 ± 0.59 3.14 ± 2.62 2.29 ± 1.65 3.22 ± 1.80 3.99 ± 2.06 1.92 ± 1.48	4.74 ± 3.54 6.28 ± 1.32 2.43 ± 1.11 2.18 ± 0.47 3.49 ± 1.01 0.37 ± 0.13 2.32 ± 0.42 1.32 ± 0.70 3.56 ± 3.02 3.66 ± 2.55 7.40 ± 5.10 4.40 ± 2.97 4.15 ± 3.56	34.046 10.574 0.191 0.002 26.264 0.265 0.012 0.731 13.188 14.343 160.690 45.384 104.295	<0.001 <0.001 0.819 0.397 <0.001 <0.001 0.575 <0.001 0.121 <0.001 <0.001 0.100 <0.001

BMI, body mass index; FT4, free T4; TSH, thyroid stimulating hormone; hsCRP, serum hypersensitive C-reactive protein; TC, total cholesterol; TG, triglyceride; HDL, high-density lipoprotein; LDL, low-density lipoprotein; FFA, free fatty acid; ApoA1, apolipoprotein A1; ApoB, apolipoprotein B.

p: p values of patients in the Control group and Hypothyroidism group.

p < 0.05 indicates statistical significance.

**Table 4:** Comparison of FT4, TSH levels and levothyroxine sodium tablets between SIBO positive and SIBO negative pregnant women with clinical hypothyroidism during pregnancy.

Hypothyroidism Group (n = 231)				
parameters	SIBO+ (n = 112)	SIBO- (n = 119)	F	P-value
FT4 (pmol/L)	11.58 ± 1.92	11.66 ± 1.66	3.909	0.753
TSH (mIU/L)	2.40 ± 1.51	2.49 ± 1.64	1.28	0.850
LT4 (ug/d)	50.89 ± 25.54	36.97 ± 18.65	1.757	<0.001

FT4, free T4; TSH, thyroid stimulating hormone; LT4, levothyroxine.

p < 0.05 indicates statistical significance.

**Figure 2:**
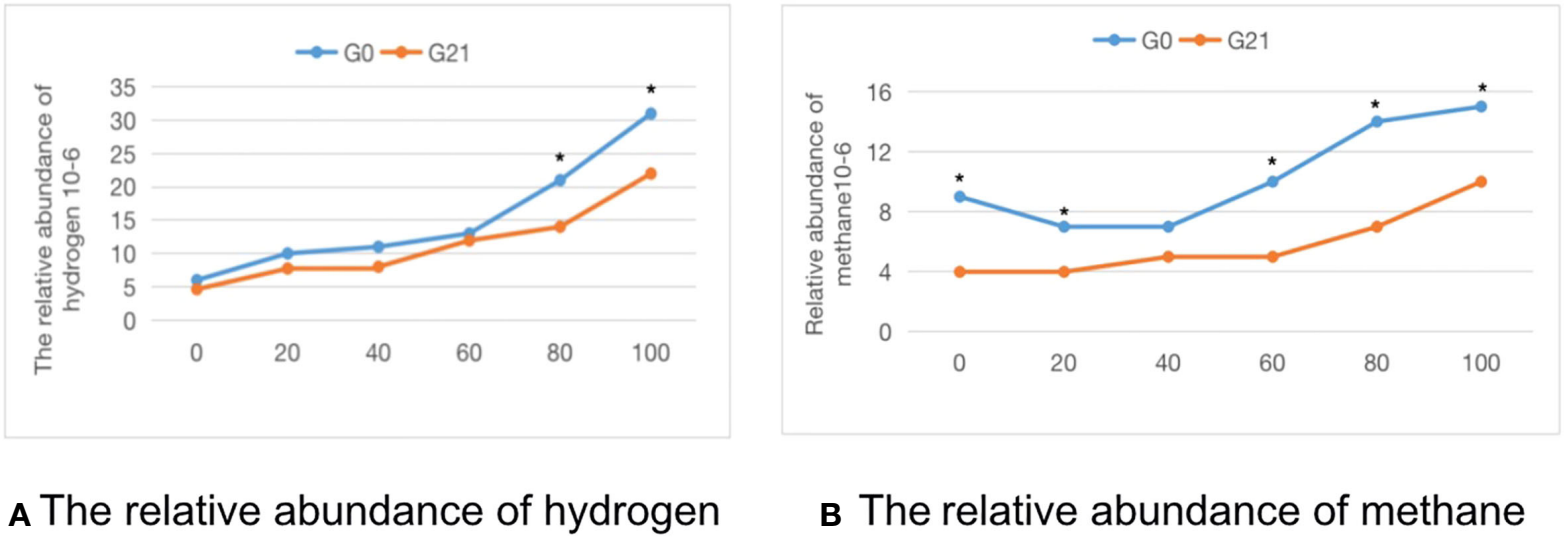
The time-abundance curve of hydrogen and methane in the G0 group and G21 group. **(A)** The relative abundance of hydrogen. **(B)** The relative abundance of methane. *indicates statistical significance.

Comparison of the methane-hydrogen breath test in pregnant patients with clinical hypothyroidism combined with SIBO positivity after 21 days of probiotics combined with prebiotics: As shown in **Table 5**, after 21 days of treatment, 46.4% of patients with clinical hypothyroidism in pregnancy combined with SIBO-positive patients turned negative, and the incidence of methane-positive patients was 3.6%, which was significantly lower than before treatment (P < 0.05). The average abundance of hydrogen and methane at each time point in The methane-hydrogen breath test after treatment was lower than that before treatment, there were significant differences in hydrogen at 80 and 100 time points and methane at 0, 20, 60, 80 and 100 time points (**Figure 2**).

**Table 5:** Positive rates of pure hydrogen, pure methane and hydromethane in the G0 and G21 groups.

	G0	G21	p
**Hydrogen-methane breath test**			
SIBO+ (%)	112	52 (46.4)	–
hydrogen+ (%)	57 (50.9)	43 (38.4)	0.060
methane+ (%)	43 (38.4)	4 (3.6)	<0.001
hydrogen+methane+ (%)	12 (10.7)	5 (4.5)	0.077
**gastrointestinal symptoms**			
diarrhea (n/%)	17 (15.2)	14 (12.5)	0.562
abdominal distension (n/%)	27 (24.1)	23 (20.5)	0.521
constipation (n/%)	49 (43.8)	30 (26.8)	0.008
No obvious symptoms (n/%)	19 (17.0)	45 (40.2)	<0.001

P0-21 P values of patients before treatment (G0) and 21 days after treatment (G21).

p<0.05 indicates statistical significance.

Changes in gastrointestinal symptoms after 21 days of probiotics combined with prebiotics: As shown in **Table 5**, the incidence of constipation after 21 days of probiotics combined with prebiotics was 30%, significantly lower than before treatment. The probability of no obvious gastrointestinal symptoms was 40.2%, significantly higher than before treatment (P < 0.05).

Comparison of clinical indicators of pregnant patients with clinical hypothyroidism combined with SIBO positivity before and after 21 days of treatment with probiotics combined with prebiotics: In **Table 6**, after 21 days of probiotics combined with prebiotics, the levels of TSH, hsCRP, IL-6, TNF-α, TC and LDL were significantly reduced compared with those before treatment (P < 0.05).

**Table 6:** Parameter changes after 0 (G0) and 21 (G21) days of treatment.

parameters	G0	G21	P	95%CI
FT4 (pmol/L)	11.58 ± 1.92	11.39 ± 2.28	0.363	-0.22~0.61
TSH (mIU/L)	2.40 ± 1.51	1.72 ± 0.61	<0.001	0.07~0.73
hsCR (mg/L) TC (mmol/L) TG (mmol/L) HDL (mmol/L) LDL (mmol/L) FFA (mmol/L) ApoA1 (g/L) ApoB (g/L) IL-2 IL-10 IL-6 IL-4 TNF-α LT4 (ug/L)	5.16 ± 3.71 6.31 ± 1.28 2.52 ± 1.14 2.23 ± 0.47 3.47 ± 1.01 0.37 ± 0.14 2.37 ± 0.41 1.28 ± 0.72 3.09 ± 2.86 3.76 ± 2.74 6.28 ± 4.40 3.92 ± 3.07 4.13 ± 3.59 50.89 ± 25.54	3.33 ± 1.71 5.56 ± 0.75 2.30 ± 1.08 2.11 ± 0.51 2.57 ± 0.57 0.36 ± 0.13 2.37 ± 0.41 1.40 ± 0.72 2.80 ± 2.35 3.76 ± 2.74 4.09 ± 2.60 4.20 ± 3.03 2.67 ± 2.61 48.88 ± 23.10	<0.001 <0.001 0.161 0.063 <0.001 0.526 0.924 0.428 0.990 0.929 <0.001 0.532 0.003 0.538	1.23~2.44 0.49~1.05 -0.09~0.51 -0.01~0.25 0.69~1.13 -0.20~0.04 -0.12~0.11 -0.28~0.12 -0.43~1.01 -0.74~0.73 1.12~3.26 -1.17~0.61 0.44~2.05 -4.40~8.42

FT4, free T4; TSH, thyroid stimulating hormone; HsCRP, serum hypersensitive C-reactive protein; TC, total cholesterol; TG, triglyceride; HDL, high-density lipoprotein; LDL, low-density lipoprotein; FFA, free fatty acid; ApoA1, apolipoprotein A1; ApoB, apolipoprotein B; LT4, levothyroxine.

P: p values of patients in the G0 group and G21 group.

p < 0.05 indicates statistical significance.

## Discussion

In recent years, studying the intestinal microbiome has become a popular field. Some studies have shown that the intestinal microbiome is related to thyroid function, and its balance is of great significance to the stability of the human body, especially the function of the endocrine system (Zhang et al., 2019). Intestinal bacterial overgrowth (SIBO) is characterized by gastrointestinal symptoms resulting from the abnormal proliferation of bacterial species in the small intestine, mainly including Gram-negative aerobic and anaerobic bacteria, which can ferment gas-producing carbohydrates (Shanab et al., 2011; Pimentel et al., 2020). However, how to treat SIBO-positive pregnant women with clinical hypothyroidism during pregnancy is still unclear.

Our study found that the SIBO-positive rate during pregnancy was higher in the clinical hypothyroidism group than in the normal group. The dose of levothyroxine sodium in SIBO positive patients was higher than SIBO negative patients in the clinical hypothyroidism group during pregnancy, which further indicated that hypothyroidism during pregnancy is closely related to intestinal bacterial overgrowth. The results of *Lauritano et al. (* Lauritano et al., 2007) are consistent with our findings that nongestational hypothyroidism is closely related to bacterial overgrowth in the small intestine. Studies have found that nongestational hypothyroidism and intestinal flora may interact by slowing gastrointestinal motility, reducing the expression of the sodium-iodine cotransporter (NIS) and affecting the absorption of iodine (Ebert, 2010; Knezevic et al., 2020). We believe the possible mechanism is that smooth muscle dysfunction and gastric acid secretion are decreased in patients with clinical hypothyroidism during pregnancy. With the weakening of gastrointestinal motility, the ability of intestinal bacteria removal is impaired, promoting the occurrence of intestinal bacterial overgrowth. Meanwhile iodine uptake in the gastrointestinal tract is mediated by the sodium-iodine cotransporter (NIS). When intestinal bacteria are disturbed, NIS expression may decrease, and iodine uptake may be affected, thus leading to the occurrence of gestational hypothyroidism.

Through the methane-hydrogen breath test, we found that the incidence of pure methane-positive pregnant women in the pregnancy clinical hypothyroidism group was significantly higher than that in the normal group, and the incidence of abdominal distention and constipation in the pregnancy clinical hypothyroidism group was also higher than that in the normal group. Hydrogen-producing bacteria are mainly *Clostridium, Enterobacter, Klebsiella* and *Bacillus*, and *Methanobrevicter Smithii* and *Methanosphaera Stadtmanae* have also been identified as the main methanogens in the human intestinal tract (Su et al., 2018). Previous studies conducted by our research group on fecal 16S RNA sequencing found that hydrogen-producing *Clostridium* was relatively enriched in the intestinal flora of patients with clinical hypothyroidism during pregnancy, which was inconsistent with the results of our methane-hydrogen breath test. This fact may be due to the sample size. *Lepp et al.* believed that methanogens consume short-chain fatty acids when producing methane (Lepp et al., 2004). Meanwhile, studies have confirmed that short-chain fatty acids can stimulate the expression of thyroid hormone, and the ability of intestinal flora to produce short-chain fatty acids in patients with hypothyroidism is significantly decreased (Su et al., 2020). Therefore, we believe that the excessive growth of intestinal bacteria in patients with clinical hypothyroidism during pregnancy may increase the abundance of methanogens and lead to a decrease in the level of short-chain fatty acids, promoting the occurrence of clinical hypothyroidism during pregnancy. The increase in progesterone during pregnancy may inhibit the release of gastrin and slow gastrointestinal peristalsis, leading to constipation. Meanwhile, hypothyroidism leads to an increase in the abundance of methanogens, slowing down intestinal transport time and causing constipation and abdominal distension.

In this study, we found that the levels of hsCRP, IL-10, IL-6 and TNF-α in pregnant women with clinical hypothyroidism during pregnancy were significantly higher than those in the control group. *Tang C et al.* also believed that hypothyroidism was related to inflammatory factors, including IL-6, TNF-α and hs-CRP, and the concentration of inflammatory factors was relatively high in nonpregnant hypothyroidism patients (Abbas and Sakr, 2016; Tang et al., 2021), consistent with our research results. Our previous studies on fecal 16S RNA sequencing found that increased CRP was related to *Gammaproteobacteria, Pasteurellaceae* and *Firmicutes*, and the combined effect of CRP and phosphocholine on bacteria could activate inflammatory factors. Intestinal leakage is an important mechanism leading to the inflammatory state of the body. Intestinal epithelial cells are connected by tight junctions and seal cell bypass to act as a selective osmotic barrier (Zheng et al., 2021). When intestinal leakage occurs, disturbance of intestinal flora may lead to an increase in intestinal permeability, bacterial translocation, and the abundance of inflammation-related flora, such as *Gammaproteobacteria* and *Pasteurellaceae*. The accumulation of harmful metabolites of the flora in the intestine further activates the inflammatory response. Aggravating tissue damage affects the metabolism of thyroid follicular cells, leading to thyroid hormone synthesis disorder and clinical hypothyroidism during pregnancy.

The concentrations of TC, LDL, FFA and ApoB in pregnant women with clinical hypothyroidism were significantly higher than those in the control group. Many studies have reported that elevated TC and LDL levels are a significant feature of hypothyroidism (Zhu and Cheng, 2010; Peppa et al., 2011). According to the study of *Jung et al.* the decrease in the number of LDL receptors in the serum of nonpregnant hypothyroidism patients reduced the LDL clearance rate, resulting in an increase in LDL and ApoB levels (Jung et al., 2017). Our previous studies on lipid metabolism (Li et al., 2021) found that the infectious pathway of pathogenic Escherichia coli was significantly higher in the disease group than in the normal group, which may be related to the increased level of phosphatidylethanolamine (PE) in hypothyroidism patients during pregnancy. And PE can maintain the stability of the cell membrane, affect the function of membrane proteins, and stimulate the occurrence of inflammation. Changes in gut microbiome composition and function as a result of inflammation are markers of metabolic damage, and its metabolites, such as lipopolysaccharides and endotoxins, may reduce the integrity of cellular connections (Farzi et al., 2018). Therefore, we speculate that when clinical hypothyroidism occurs in pregnancy, the pathogenic Escherichia coli metabolic pathway is dominant, accompanied by increased PE levels, resulting in intestinal microflora disorder in pregnant women, reducing the stability of cell connections and reducing the number of LDL receptors, LDL, TC, ApoB and other lipid synthesis and degradation disorders.

After 21 days of probiotics combined with prebiotics, 46.4% of SIBO-positive pregnant women with clinical hypothyroidism in pregnancy turned negative, and the pure methane positive rate was significantly lower than before the treatment, but treatment after 21 days significantly reduced the expiratory hydrogen methane experiment and the average abundance of hydrogen and methane at all time points and significantly improved the patients with constipation. *Pimentel et al.* believe that probiotics can improve the composition of intestinal microbes in a beneficial way and relieve gastrointestinal symptoms of constipation (Pimentel et al., 2006; Zhang et al., 2010; Nickles et al., 2021), which is consistent with our research results. *Audrey et al.* did not find that the combination of probiotics and prebiotics had a significant effect on improving gastrointestinal symptoms in nonpregnant healthy subjects (Talebi et al., 2020), which is different from our study. This finding may be related to different factors, such as the study population, disease and treatment duration. We think that the effective treatment of probiotics and prebiotics may increase the number of *lactobacilli* and *Bifidobacterium* in the intestinal tract, reduce adverse metabolic products of gut microbes, and increase the content of short-chain fatty acids and methyl acetate to maintain the balance of intestinal flora and small intestine bacterial overgrowth. In addition, the short-chain fatty acids produced by probiotics in intestinal fermentation lead to osmotic stimulation, which can improve the frequency of intestinal peristalsis and stool characteristics, thus improving gastrointestinal symptoms such as constipation. At the same time, probiotics combined with prebiotics may improve the abundance and diversity of intestinal methanogens in patients with clinical hypothyroidism combined with SIBO positivity during pregnancy and improve constipation symptoms by reducing the production of intestinal methane and speeding up intestinal operation time.

Thyroid function of patients with clinical hypothyroidism complicated with SIBO during pregnancy was controlled within the normal range before and after treatment, and TSH level decreased within the normal range after 21 days of treatment compared with before treatment. *Sepide et al. (* Talebi et al., 2020) believed that the combination of probiotics and prebiotics did not significantly improve thyroid function. We found that this difference may be related to the sample size and the amount of oral levothyroxine sodium in patients. As a part of the intestinal barrier, the intestinal microbiota not only regulates the tight connection of cells and intestinal permeability but also regulates the characteristics and mucus components of intestinal epithelial cells. When the intestinal microbiota is disturbed, the absorption of thyroid hormones is significantly affected (Virili and Centanni, 2015). Our previous studies found that the abundance of *Roseburia, Pasteurellales, Lachnospira* and other bacteria in the intestinal flora of patients with clinical hypothyroidism during pregnancy was high, and the metabolites of these bacteria were often harmful to the body. Studies have shown that probiotics combined with prebiotics can improve the intestinal flora disturbance, maintain the stability of the intestinal permeability and maintain the TSH level (Sergeev et al., 2020; Mohamed et al., 2021). *Lactobacillus-* and *Bifidobacterium-related* bacteria can protect intestinal epithelial cells from the gut microbes’ harmful metabolites qualitative damage, so probiotics combined with prebiotics may increase the abundance of beneficial bacteria groups, ensuring the integrity of the intestinal mucosal barrier can reduce TSH levels and improve thyroid function.

The levels of hsCRP, IL-6 and TNF-α in SIBO-positive pregnant women with clinical hypothyroidism after treatment with probiotics combined with prebiotics were significantly lower than before treatment, and the concentrations of TC and LDL were also lower after treatment. The combination of probiotics and prebiotics is fermented by beneficial bacteria to produce short-chain fatty acids (SCFAs), which may signal through metabolism-sensitive G-protein-coupled receptors and free fatty acid receptors in the intestinal epithelium and inhibit tissue deacetylase, thus inhibiting systemic inflammatory responses (Vinolo et al., 2011; McLoughlin et al., 2017). Meanwhile, SCFA can reduce cholesterol in the blood by blocking the synthesis of cholesterol in the liver (Thandapilly et al., 2018). We believe the possible mechanism is that the combination of prebiotics and probiotics may regulate the disorder of intestinal flora, inhibit the increase in harmful flora, stabilize the integrity of the intestinal mucosal barrier, and thus downregulate inflammatory factors such as IL-6 and TNF-α. And the use of probiotics combined with prebiotics may increase the abundance of beneficial bacteria such as Bifidobacterium and Lactobacillus in the intestinal flora, which stimulate the production of SCFA, which in turn acts as a ligand to activate peroxisome proliferator-activated receptor (PPAR), which reduces the synthesis and absorption of LDL and TC.

### Strengths and limitations of the study

The advantages of this study is that the methane-hydrogen breath test was used in this study to evaluate the intestinal microflora of patients with clinical hypothyroidism during pregnancy. This method is convenient, noninvasive and fast for evaluating the overgrowth of intestinal bacteria, and it provides a new idea for the treatment of patients with clinical hypothyroidism during pregnancy combined with SIBO positivity. However, some limitations of this study must be acknowledged, such as the relatively short follow-up period, and some insignificant changes may be statistically significant with the extension of follow-up time. In addition, in our study, only the methane-hydrogen breath test was used to evaluate the changes in intestinal flora in patients with clinical hypothyroidism combined with SIBO positivity during pregnancy, which had certain limitations. We will expand the sample size and further study the changes in intestinal flora by using 16S RNA sequencing technology.

## Conclusion

In conclusion, probiotics combined with prebiotics can not only improve the overgrowth of intestinal bacteria but also adjust thyroid function and have a certain controlling effect on the body’s inflammatory response, providing new ideas for the treatment of clinical hypothyroidism combined with intestinal bacterial overgrowth during pregnancy.

## Data availability statement

The original contributions presented in the study are included in the article/Supplementary Material. Further inquiries can be directed to the corresponding author.

## Ethics statement

The studies involving human participants were reviewed and approved by the Ethics Committee of the Third Affiliated Hospital of Zhengzhou University. The patients/participants provided their written informed consent to participate in this study.

## Author contributions

Conception and design: YX and YH. Collection and assembly of data: YH, YB, JL, YC, QO, and BW. Data analysis and interpretation: YH, ZS, MZ, and YB. Manuscript writing: YX and YH. All authors contributed to the article and approved the submitted version.

## Funding

This research was funded by Henan provincial science and technology research and development special funds #182102410020 (YX).

## Acknowledgments

We gratefully acknowledge our patients for their voluntary participation in this study.

## Conflict of interest

The authors declare that the research was conducted in the absence of any commercial or financial relationships that could be construed as a potential conflict of interest.

## Publisher’s note

All claims expressed in this article are solely those of the authors and do not necessarily represent those of their affiliated organizations, or those of the publisher, the editors and the reviewers. Any product that may be evaluated in this article, or claim that may be made by its manufacturer, is not guaranteed or endorsed by the publisher.
